# Community Perceptions of Blackfly Nuisance and Acceptability of the “Slash and Clear” Vector Control Approach in the Ntui Health District of Cameroon: A Qualitative Study

**DOI:** 10.3390/ijerph21060658

**Published:** 2024-05-22

**Authors:** Joseph Nelson Siewe Fodjo, Serge Raoul Ekukole Ekome, Julius Ndi Taryunyu Njamnshi, Wepnyu Yembe Njamnshi, Kongnyu G. Njamnshi, Leonard Ngarka, Alfred K. Njamnshi

**Affiliations:** 1 Brain Research Africa Initiative (BRAIN), Yaoundé P.O. Box 25625, Cameroon; ekukssquare@yahoo.com (S.R.E.E.); njamjulius@gmail.com (J.N.T.N.); wepnyu.njamnshi@brainafrica.org (W.Y.N.); kongnyu.njamnshi@brainafrica.org (K.G.N.); lngarka@yahoo.com (L.N.); 2 Global Health Institute, University of Antwerp, Doornstraat 331, 2610 Antwerp, Belgium; 3 Division of Operational Research in Health, Ministry of Public Health, N°8 Rue 3038 quartier du Lac (Yaoundé III), Yaoundé P.O. Box 3595, Cameroon; 4 Neuroscience Lab, Faculty of Medicine and Biomedical Sciences, The University of Yaoundé 1, Yaoundé P.O. Box 1364, Cameroon; 5 Neurology & Clinical Neuroscience Department, Yaoundé Central Hospital, Yaoundé P.O. Box 87, Cameroon

**Keywords:** onchocerciasis, blackfly, qualitative study, slash and clear, Cameroon

## Abstract

Besides being vectors of the onchocerciasis parasite, blackflies are a source of nuisance in onchocerciasis-endemic communities. We investigated the experience of residents in the Ntui Health District (Cameroon) regarding blackfly nuisance and assessed their perceptions of a novel “Slash and Clear” (S&C) intervention for blackfly control. Focus group discussions were conducted before and after S&C implementation (respectively, in February 2022 and December 2023). Blackflies were known to emerge from the river areas and cause disease. To prevent blackfly bites, the population often covered their body with protective clothing and applied various substances (kerosene, oil, or lemon) to their skin. Post-intervention data showed reduced blackfly nuisance, and the willingness to sustain blackfly control in the long-term was unanimous among community leaders and members, including the village volunteers who implemented the S&C intervention. In conclusion, blackfly nuisance is evident in the Ntui onchocerciasis focus of Cameroon and led to a panoply of coping practices, some of which could be detrimental to their health. Implementing S&C for blackfly control is well accepted and could sustainably alleviate the nuisance caused by blackflies while simultaneously breaking the onchocerciasis transmission cycle.

## 1. Introduction

The neglected tropical disease onchocerciasis is caused by the filarial parasite *Onchocerca volvulus* and transmitted by blackflies (Diptera: *Simuliidae*), which breed in fast-flowing fresh waters [1]. It is estimated that 99% of the 20.9 million persons with onchocerciasis worldwide live in Africa [1]. While onchocerciasis is mostly known for its ocular symptoms, which can lead to blindness (also known as “river blindness”), its hallmark clinical presentation includes skin disease manifesting as troublesome itching and clinical lesions such as acute papular onchodermatitis, chronic papular onchodermatitis, lichenified onchodermatitis, skin atrophy, depigmentatation (“leopard skin”), and subcutaneous nodules [2,3]. Accumulating evidence also suggests neurological manifestations of onchocerciasis, notably onchocerciasis-associated epilepsy (OAE) [3,4]. In view of its clinical presentation (particularly the disfiguring skin lesions), onchocerciasis is considered a stigmatizing condition [5,6], and this engenders significant psychosocial stress in the affected individuals. Indeed, it is known that onchocerciasis negatively impacts the quality of life of infected individuals [7]. In addition, blackfly vectors are a source of nuisance in communities located close to breeding sites, notably via their painful/itchy bites and resulting skin marks [8,9]. 

Onchocerciasis is still widely endemic in Cameroon, where the rich hydrographic networks with frequent rapids and dams provide ideal conditions for blackfly breeding. *Simulium* species identified as potent *O. volvulus* vectors in Cameroon include the *Simulium damnosum* complex (*S. damnosum* sensu stricto, *S. sirbanum*, *S. yahense*, and *S. squamosum*), with a new variant of *S. squamosum E* recently identified in the Mbam valley [10]. Previous entomological studies along the Sanaga and Mbam rivers of Cameroon have described several blackfly breeding sites [10,11,12,13]. Concurrently, there have been reports of the nuisance caused by these insects in the nearby villages [14,15]. In the early 1990s, an entomological assessment revealed extremely high blackfly biting rates in Cameroon, reaching 8000 bites per man per day in some sites [16]. This blackfly nuisance prompted the implementation of various blackfly control techniques in the affected zones. 

Since adult blackflies are the source of the nuisance, one would think that destroying these mature insects is the course of action. However, this approach has proven to be difficult (if not impossible) because of the great dispersal of adult blackflies and the difficulties of tracking their niches when they are not flying around in search of a blood meal [17]. Therefore, the common practice is to attack the larval developmental stage of the blackflies, as the latter are clustered in well-defined breeding sites. Killing larvae should result in fewer blackflies in the next generation, while the current generation of adult vectors would be expected to die within four weeks [18]. 

The first larvicidal chemical that was initially used in Cameroon was a powder form of dichlorodiphenyltrichloroethane (DDT), which was sprayed over the infested rivers [16]. However, despite its great efficacy and low cost, DDT was discontinued after an expert report highlighted the environmental dangers of its decomposition products, which continued to accumulate after the larviciding sessions [16]. By 1977, DDT was completely replaced with an emulsifiable concentrate of temephos, a very active product with reduced toxicity, rapid biodegradability, and 100% effectiveness at more than 50km downstream of the application spot on the river. Temephos initially worked properly, until the emergence of blackfly resistance to this product was observed in the Cameroonian setting [19]. In addition, the application of temephos is costly, logistically demanding, and requires specific expertise. It became evident with time that a non-chemical and easily implementable blackfly control technique would be ideal to ensure sustainability and avoid deleterious environmental consequences. 

In this regard, an environmentally friendly approach for blackfly control was developed and has been tested in different settings. This so-called “Slash and Clear” method is a community-directed vector control technique that entails the identification and physical destruction of blackfly breeding sites using machetes. When applied in a given area (either once or repeatedly each year), “Slash and Clear” can substantially reduce the blackfly population and consequently lower the biting rates. During pilot investigations in Uganda, implementing “Slash and Clear” on a small river resulted in significantly lower biting rates post-intervention [20]. In addition, “Slash and Clear” has been shown to be a cost-effective intervention that could accelerate onchocerciasis elimination even in resource-limited settings [21]. “Slash and Clear” activities were recently conducted along the Mbam River in Cameroon by Domche et al., who also observed significant reductions in biting rates [22]; however, they did not document the population’s perception or acceptability of this new technique.

Our research team has been conducting a research project for the past three years, aiming to implement the “Slash and Clear” approach along segments of the longest river in Cameroon, the Sanaga River, and investigate community perceptions as well as the sustainability of this intervention [23]. Thus, the first phases of the project consisted of formative research, whereby qualitative studies were conducted to understand how the community experienced the blackfly nuisance and whether the “Slash and Clear” intervention was welcomed in the study villages. We were also interested in achieving community ownership and sustainability of the “Slash and Clear” intervention in the study villages. Therefore, we also conducted post-intervention qualitative investigations to understand the community’s views regarding the long-term implementation of blackfly control using the “Slash and Clear” approach. Indeed, according to mathematical models, sustainable blackfly interventions represent an important step toward achieving the elimination of transmission of onchocerciasis in the endemic sites [24].

## 2. Materials and Methods

### 2.1. Study Setting

We conducted the study in two phases: pre-intervention (February 2022) and post-intervention (December 2023). The intervention consisted of two rounds of “Slash and Clear”, implemented, respectively, in March 2023 and December 2023. The selected study sites were three villages of the Ntui Health District (Centre Region, Cameroon), namely: Nachtigal, Essougli, and Ndowe. These study sites have been extensively described elsewhere, with evidence of ongoing onchocerciasis transmission [12]. Onchocerciasis control using annual ivermectin mass drug administration has been implemented for over two decades in the Ntui Health District, but the coverage has been low (<60%) in the study villages [25]. The study area is a rural setting with forest-like vegetation, traversed by the Sanaga River, where blackfly breeding sites have been identified [12]. The most common livelihood activity of the study population is agriculture, both subsistence crops and cash crops (mainly cocoa). Entomological studies conducted in Nachtigal and Essougli villages during the pre-intervention phase (before initiation of “Slash and Clear”) found that the median monthly blackfly biting rates ranged from 14,366 to 34,477 per man [12]; these numbers were halved after the implementation of “Slash and Clear” (post-intervention phase) [26], translating to a reduced blackfly abundance in those villages. 

### 2.2. Study Design 

This was a qualitative study that consisted of five focus group discussions (FGDs) and three semi-structured interviews (SSIs). 

### 2.3. Study Participants and Sampling Procedure 

We started by conducting community engagement visits in all the villages. This entailed initiating contact with the village authorities and explaining to them that our project seeks to better understand the onchocerciasis and blackfly situation in their communities, and this will require that we have discussions or interviews with some of them. Thereafter, we invited the village chiefs and selected adult community members for a meeting during which the purpose and methodology of the FGD were explained, including the need to audio-record the conversations. Those who provided verbal consent were then retained for the FGD proper. The constitution of each FGD was created purposively and by convenience, according to each participant’s availability and willingness to participate. At the end, each FGD had between 9 and 12 participants. Also, in each village, one in-depth SSI was conducted with the chief. 

### 2.4. Data Collection

Data were collected in two phases: The pre-intervention phase in February 2022 (before “Slash and Clear”) consisted of one FGD in each of the three study villages, plus one SSI with each village chief. The post-intervention phase added two more FGDs, one in each of the two villages where the intervention had been implemented (Nachtigal and Essougli). These follow-up FGDs included some of the village volunteers involved in the “Slash and Clear” activity, in addition to the village chiefs and other community members.

For both the pre-intervention and post-intervention phases, the research team encountered the participants at an agreed time and place within the village. All participants sat around a table in a quiet room or outdoor space; the audio recording was initiated, and explanations regarding the FGD/SSI activity were provided once more for all individuals present to freely consent verbally to participate. A trained social scientist (SE) moderated the discussions during both the FGDs and SSIs, and ensured that the participants remained within the study context in their responses. All participants were versed in oral French; therefore, we conducted all discussions and interviews in the French language. At the end of each FGD/SSI, the moderator thanked the participants, and the entire research team addressed any remaining questions or concerns from them. 

### 2.5. Data Analysis 

Data from the FGDs and SSIs were transcribed verbatim by the social scientist (SE). Subsequently, a thematic analysis was conducted. The lead author (JNSF) and the social scientist (SE) carefully read the transcripts and identified relevant themes. Codes were created under the different themes to systematically regroup the participants’ views. Relevant data from participants were summarized to fit within given codes under a specific theme. This process was influenced by the original research interests (understanding perceptions regarding blackfly nuisance and vector control using the “Slash and Clear” approach), as well as concepts generated inductively from the data as described by Pope et al. [27]. Quotes selected for the article were translated into English by the lead author and other co-authors (JTN, KGN, and WYN) who were involved in the data collection phase.

### 2.6. Ethical Considerations

Ethical approval for the study was obtained from the institutional review board of the Cameroon Baptist Convention Health Board (Ref: IRB2021-03), and a research permit for the project was granted by Cameroon’s Ministry of Scientific Research and Innovation (Ref: 000144/MINRESI/B00/C00/C10/C13). Participation in the FGDs/SSIs was totally voluntary, and informed verbal consent was obtained from all participants in the study. The audio recording device containing the study recording was securely stored in an office under lock-and-key, and the recordings were accessed only by the social scientist for transcription purposes, during which all personal information was removed. Respondents were not paid for their participation in the study.

## 3. Results

Data from the five FGDs and three SSIs were analyzed. The local word for blackflies in the study communities was “*mout mout*”. We focused on five main themes that seemed relevant for the perceptions and experiences of the participants vis-à-vis blackflies: “blackfly origin”, “blackfly nuisance”, “coping mechanisms”, “preventing blackfly bites”, and “Slash and Clear intervention”. The relevant themes and codes that emerged from our data are summarized in Table 1.

### 3.1. Blackfly Origin

During the FGDs, the predominant opinion was that the blackflies emerged from trees and shrubs. Only one participant (from Nachtigal) mentioned that blackflies originate from the river.

*“Those small plants at the river… that is where the ‘mout mout’ come from”.*

(FGD participant, Nachtigal).

*“Because there are some trees here, the ‘iriko’… the ‘mout mout’ stay there, and many of them come from there”*

(FGD participant, Essougli).

### 3.2. Blackfly Abundance and Nuisance

All participants admitted to having a high abundance of blackflies in their villages. The word “black” was repeatedly used to give a visual picture of a body surface (hands and legs) covered with many blackflies. Blackflies reportedly abound everywhere in the villages, although some participants specified that it is worse at the riverside.

*“Mout mout are very abundant at the river. But they are everywhere”.*

(FGD participant, Essougli).

*“Here, the mout mout can be found everywhere… Everybody can be seen beating himself (to kill the blackflies on their body)”*

(FGD participant, Ndowe).

*“Sir, there is no quarter in this village that does not have mout mout…”*

(FGD participant, Nachtigal).

Regarding the periodicity of blackflies, they were found to bite mostly in the morning and evening hours. Also, participants reported that blackflies are more abundant in the rainy season compared to the dry season.

*“Oh yes. They [blackflies] bite a lot in the morning and in the evening. And when the rains start, it gets bad… October is the worse!”*

(FGD participant, Ndowe).

*“You get out in the morning, after just 30 minutes… your legs are all black with mout mout”.*

(FGD participant, Essougli).

The nuisance caused by blackfly bites was also evident, as the participants were emphatic about the discomfort that these insects cause, be it in the morning or the evening. This has led to significant disruptions in their daily routines.

*“When it comes to using the [external] toilets, we must do it very early in the morning before 6am. When day light arrives, it becomes impossible to use the toilets because of the mout mout”.*

(FGD participant, Essougli).

*“Sometimes even to take a bath, you are afraid”.*

(FGD participant, Essougli).

*“You see, many people want to go the farm, but they have to wait till around eight o’clock to start working there. Because if you go there too early, when you bend down to work the farm and you are talking at the same time, you will swallow a good amount of blackflies”.*

(FGD participant, Ndowe).

Both the FGDs and the SSIs with the village chiefs revealed that there has been no major population displacement or abandonment of land near the river because of the blackflies.

### 3.3. Practices Regarding Blackflies

The participants highlighted a few coping mechanisms that the villagers commonly use to counter the blackfly problem in their community and prevent the painful or itchy bites from these insects. These included the following: wearing long clothing to cover all skin surfaces; rubbing their skin with lemon, palm oil, engine oil, or kerosene; sleeping under a mosquito net; using smoke to drive blackflies; and using chemical insecticides (commercially available) inside their homes. There was also a belief by some participants that the intake of ivermectin (a drug distributed yearly during mass drug administration campaigns for onchocerciasis elimination) would protect them from blackflies.

*“People here only have to wear trousers and socks… Some people even wear gloves”.*

(FGD participant, Ndowe).

*“Some ways to fight against mout mout: Lighting a fire in the house or in the farm to drive them by the heat… Rubbing kerosene or engine oil on my body to drive them with the smell… Rubbing lemon on my skin”.*

(FGD participant, Nachtigal).

*“Traditionally, we often burn the chaffs of palm nuts to produce smoke that will drive away the mout mout… but they [blackflies] return once the smoke is finished”.*

(FGD participant, Ndowe).

*“When you do not have mosquito net, some people use Moon Tiger [commercial insecticide] to calm the situation at home… but this thing is toxic for our health”.*

(FGD participant, Essougli).

However, a few participants reported that most of the common practices against blackflies were no longer effective and/or that blackflies had already adapted to them.

*“Sir, even some of these long trousers women wear… sometimes when they come back home in the evening, we still see marks [of blackfly bites] on their legs”.*

(FGD participant, Ndowe).

*“Those [mout mout] of now have already adapted [to our strategies]…”*

(FGD participant, Nachtigal).

### 3.4. Perceptions of the “Slash and Clear” Vector Control Approach 

In all three villages, there was a very positive pre-intervention perception of the “Slash and Clear” strategy, although the villagers had never heard about it before. Considering the suffering inflicted by the blackflies, they were willing to adopt any strategy that could produce results.

*“It has been many years, we don’t even know what to do. If we have to go down to the river, the community is ready to follow you and do whatever is needed”.*

(FGD participant, Nachtigal).

*“The first thing is to get the equipment [for Slash and Clear]. Once we have the necessary equipment, the rest will follow easily”.*

(FGD participant, Essougli).

*“There are very active youths here… who will be very willing to do the work”.*

(FGD participant, Ndowe).

### 3.5. Feedback from Volunteers Involved in “Slash and Clear” Implementation at the River

While acknowledging that the intervention substantially reduced blackfly nuisance in their respective villages, the “Slash and Clear” volunteers highlighted several difficulties encountered during its execution. Notably, the activity was viewed as being physically demanding and risky, as some volunteers reported the obstacles they encountered while clearing vegetation at the breeding sites.

*“Yes, we can see that the abundance of mout mouts is decreasing. Before you [the research team] came here, they were many but since we started the work we can see fewer bites”.*

(Volunteer, Nachtigal).

*“There were big difficulties on ground… especially because the places where we must reach to cut the grass on the river are very delicate”.*

(Volunteer, Nachtigal).

*“To go far into the river… even with the life jacket, I was no longer myself [I was very scared]… It is very difficult and risky”.*

(Volunteer, Essougli).

*“For example, one day I was working there with my colleague and we met a snake. You see, if you begin to shake you can even fall in the water and drown”.*

(Volunteer, Nachtigal).

Following the implementation of “Slash and Clear” by the village volunteers, there was an overall willingness to continue this intervention by themselves because it greatly helped to reduce the blackflies in their community. The volunteers, however, insisted that adequate equipment (machettes, boots, gloves, etc.) be provided for them to sustain the intervention in the long run. Village chiefs and other community members also attested to the reduced blackfly nuisance following the “Slash and Clear”.

*“Yes, it is very important to continue”.*

(Volunteer, Nachtigal).

*“But if we don’t have the right equipment for the work, we cannot continue doing it”.*

(Volunteer, Essougli).

*“For me, I will love that the “Slash and Clear” activity should continue… we cannot start an activity and then we leave it half way. We really need to eradicate mout mouts because there is a lot of onchocerciasis here”.*

(Chief, Essougli).

## 4. Discussion

Our findings describe how communities in an onchocerciasis focus in Cameroon perceive and experience blackflies (locally known as “*mout mout*”) and the nuisance they engender. We also document some coping mechanisms in these communities aimed at either driving away the insect or attenuating its effects on the well-being of the village residents. The communities’ views regarding blackfly control as well as their long-term expectations concerning the “Slash and Clear” approach were also captured during this study. Concerning their perceptions of the origin of blackflies, the participants in the Ntui Health District still have prevailing misconceptions. The predominant idea is that blackflies emerge from trees and shrubs, most likely because these are abundant at the riverside. However, explaining that blackflies breed in fast-flowing waters is important as it guides the community in identifying areas along the river where interventions would be most impactful. 

The participants’ responses regarding blackfly nuisance concur with the biting patterns observed in these vectors. Indeed, the complaints of more frequent blackfly bites in the morning to the point of disrupting bathing or farming activities align with the diurnal peaks for blackfly biting, notably in the morning and evening hours as observed in Akwa Ibom (Nigeria) [28], Maridi (South Sudan) [29], and in the Twifu-Heman Lower Denkyira District (Ghana) [30]. However, our findings of early morning bites contrast with observations from South Western Nigeria, where peak biting rates were observed in the middle of the day [31]. No matter the reported biting patterns, the fact that *Simulium* are day-biting insects [32] (as opposed, for instance, to *Anopheles* mosquitoes, which typically bite at night [33]) implies that they can be a nuisance to the day-time productivity of affected communities, thereby constituting a challenge to local economic development. As such, onchocerciasis is rightly classified as a disease of poverty, as it creates a vicious circle by making it difficult for the already impoverished populations to work productively during the day. Thus, eliminating onchocerciasis would have a double advantage for these villages: better health and increased economic viability [34]. 

Given that we provided the participants with information about the role of blackflies in onchocerciasis transmission as part of the information/consenting process during the FGD and SSI, we did not assess this aspect during our discussions. Notwithstanding, virtually all residents in the study villages were already aware that blackflies can transmit diseases, including “filaria” (referring to the itchy onchocercal skin disease), possibly owing to the previous onchocerciasis research projects in that area. Similar to findings from villages in the Mbam valley (Cameroon) [14], Alabameta (Nigeria) [35], and Enugu (Nigeria) [8], participants in Ntui admitted that blackfly bites occur throughout the community but more frequently near the river. As with the study by Domche et al. along the Mbam River in Cameroon [14], the rainy season was also identified as a period of more abundant blackflies and increased nuisance. This increased blackfly abundance during the rainy season concurs with the increased biting rate that was noted in our study area during the months of intense rainfall, with a peak in the month of October [12]. October was also highlighted by FGD participants as the month during which they experienced intense blackfly nuisance. Therefore, vector control interventions should target the periods preceding rainy seasons to have maximum impact on onchocerciasis transmission. 

Akin to previous findings in several onchocerciasis foci, the most frequently mentioned protective mechanism against blackflies was the use of long clothing to cover as much skin surface as possible [8,14,35]. However, there are reports of blackflies being able to enter the gaps in sleeves and lowers of trousers to bite on forearms and legs, respectively [32]. This may explain why some of our respondents complained of incurring bites despite wearing protective clothing. In addition, our study participants affirmed the common usage of topical substances (lemon, kerosene, palm oil, etc.) as reported in other settings [14,35], some of which could cause skin irritation. The practice of burning grass to produce smoke that would drive away blackflies, as observed in this study, was also evoked by villagers in Imo state, Nigeria [36]. In the two riverside study villages (Nachtigal and Essougli), it was believed that ivermectin intake also fights against blackflies. However, while it has been shown that mosquitoes that feed on ivermectin-treated individuals have reduced life expectancies [37], there is no evidence that ivermectin can affect the survival of blackflies [38]. It is plausible that the reduced itchy sensations following ivermectin intake have been misinterpreted as a reduced blackfly nuisance. Overall, the panoply of local strategies elaborated by villagers in different settings to minimize blackfly bites attest to the high level of nuisance caused by the latter, warranting urgent intervention to alleviate the population’s sufferings. 

The participants were enthusiastic regarding the implementation of a vector control intervention to reduce blackflies in their community. Despite highlighting some major difficulties and challenges with the “Slash and Clear” approach, it was well received and offers promising perspectives to accelerate onchocerciasis elimination even in places of high endemicity such as Cameroon [22,26] and South Sudan [39]. The community volunteers who actively participated in “Slash and Clear” implementation were willing to perpetuate this strategy with little or no supervision, as long as the right equipment was provided. Acceptability of the “Slash and Clear” strategy is all the more relevant because this technique is environmentally friendly [22] as opposed to the use of chemical larvicides in the river to destroy blackfly larvae. Furthermore, “Slash and Clear” is less demanding technically and can be carried out even by illiterate village volunteers. We therefore recommend that the decentralized collectivities (local councils, health actors, and community leaders) in onchocerciasis-endemic foci consider implementing routine “Slash and Clear” interventions where this is feasible. Such community ownership is crucial for the sustainability of public health interventions; indeed, even when it came to ivermectin mass drug administration, the World Health Organization opted for a community-directed approach as it proved to be effective not only for onchocerciasis but also for other health conditions afflicting the communities [40].

Our study was not void of limitations. We acknowledge the possibility that social desirability bias could have influenced the responses provided during the FGD and SSI, especially in the post-intervention studies. Indeed, as the research team funded all “Slash and Clear”-related interventions, some participants may have been uncomfortable to give any form of negative report about this vector control strategy. However, the fact that complaints regarding the practical implementation of “Slash and Clear” were raised by some volunteers attests to the wide spectrum of the responses we received. Another difficulty worth mentioning is the fact that some blackfly breeding sites were not accessible for “Slash and Clear”, implying that a proportion of blackflies would continue to thrive and onchocerciasis transmission would not be truly interrupted by the traditional “Slash and Clear” alone. Complementary measures such as optimal coverage during ivermectin mass drug administration campaigns and pinpoint larviciding of the inaccessible breeding sites (using drones, for instance) could be considered.

## 5. Conclusions

In onchocerciasis-endemic communities of the Ntui Health District in Cameroon, blackflies pose a serious nuisance to villagers via their painful bites. This has affected the quality of life of villagers, causing them to resort to various (temporary) solutions, such as rubbing kerosene, oil, or lemon on their skins, often with adverse health consequences. The “Slash and Clear” strategy was well received by the communities. Captivating local volunteers in the affected villages to implement vector control methods like “Slash and Clear” would decrease blackfly abundance, resulting in reduced blackfly nuisance and hence lower onchocerciasis transmission. We therefore recommend that public health stakeholders consider adopting this community-accepted “Slash and Clear” vector control method, which, when implemented at the right timing and in the right places, can considerably accelerate onchocerciasis elimination prospects.

## Figures and Tables

**Table 1:** Themes regarding blackfly perception and nuisance in the study villages.

Themes	Codes	Nachtigal	Essougli	Ndowe
Blackfly origin	From rivers	Yes	No	No
	From trees/shrubs/bushes	Yes	Yes	Yes
Blackfly nuisance	Bites in the farm	Yes	Yes	Yes
	Bites at home	Yes	Yes	Yes
	Bites in the morning	Yes	Yes	Yes
	Bites in the evening	Yes	Yes	Yes
	Led to population displacement	No	No	No
Coping mechanisms	Cover skin with long clothing	Yes	Yes	Yes
	Apply substance on skin	Yes	Yes	Yes
	Ivermectin intake	Yes	Yes	No
	Mosquito net	No	Yes	Yes
Preventing blackfly bites	Burn herbs/plant fibers	Yes	Yes	Yes
	Use of commercial insecticides	Yes	Yes	Yes
“Slash and Clear” intervention	Pre-intervention willingness	Yes	Yes	Yes
	Post-intervention acceptability	Yes	Yes	-
	Implementation difficulties	Yes	Yes	-
	Decreased blackfly bites	Yes	Yes	-
	Willingness to continue intervention	Yes	Yes	-

## Data Availability

The data presented in this paper are freely available through the Brain Research Africa Initiative (BRAIN) Cameroon upon reasonable request.
